# Microsurgical opening of the carotid dural rings: how I do it

**DOI:** 10.1007/s00701-025-06673-7

**Published:** 2025-10-27

**Authors:** Edgar Nathal, Alejandro Serrano Rubio, Sharon Trujillo, Rodolfo Villalobos-Díaz, Julián Moisés Enríquez-Álvarez

**Affiliations:** Department of Vascular Neurosurgery, National Institute of Neurology and Neurosurgery “Manuel Velasco Suárez”, Universidad Nacional Autónoma de México (UNAM), Av. Insurgentes Sur 3877, Tlalpan, 14269 Mexico City, México

**Keywords:** Paraclinoid aneurysm, Carotid rings, Dural rings, Keyhole approach, Surgical anatomy

## Abstract

**Background:**

Paraclinoid aneurysms represent a challenge for neurosurgeons due to the anatomical complexity of this region and the technical difficulties involved in accessing this location.

**Methods:**

In this work, we describe the surgical technique used to manage paraclinoid aneurysms, as exemplified through a clinical case. The distal dural ring (DDR) is exposed after the anterior clinoid process is resected extradurally. Then, the DDR is cut around the dorsal part of the carotid artery, moving toward the dura covering the optic nerve (ON). After, the lateral and ventral part of the ring is cut until the artery is freed from the dural attachment. The space between the proximal and DDR is visible and can be used as a proximal vascular control site if needed.

**Conclusion:**

Microsurgical clipping of paraclinoid aneurysms through a pterional or a keyhole approach, combined with a systematic technique for opening the carotid rings, is an excellent strategy for exposing and clipping paraclinoid aneurysms, yielding favorable clinical and surgical outcomes.

**Supplementary Information:**

The online version contains supplementary material available at 10.1007/s00701-025-06673-7.

## Introduction

The carotid distal dural ring (DDR) represents a critical anatomical structure formed by the circumferential fusion of the dura mater around the internal carotid artery (ICA) as it enters the intracranial compartment [7]. Meticulous dissection is essential during skull base surgery, including anterior clinoidectomy, transcavernous approach, and paraclinoid aneurysm clipping. A stepwise approach to opening the DDR is crucial to avoid catastrophic complications, such as aneurysm rupture, ICA injury, optic nerve damage, or cerebrospinal fluid (CSF) leaks.

### Relevant surgical anatomy

The carotid dural rings are intricate anatomical structures composed of two concentric fibrous layers (proximal and distal) that anchor the clinoid segment of the internal carotid artery (ICA) as it traverses the skull base [10]. Distally, the upper dural ring forms a tight collar around the ICA, demarcating the transition from the cavernous to the supraclinoid ICA. Medially, this ring creates the carotid cave, described by Kobayashi et al. in 1989, which often harbors aneurysm necks or atherosclerotic plaques [6, 7]. Proximally, the lower dural ring fuses with the carotid-oculomotor membrane, a dural sheet separating the anterior clinoid process from the oculomotor nerve (CN III). This membrane extends medially to line the optic strut's inferior surface, forming a critical boundary between the cavernous sinus and the subarachnoid space [7].

The clinoid segment of the ICA, situated between the two rings, lacks an external adventitial layer, rendering it prone to iatrogenic injury during dissection. Laterally, the dura overlying the upper ring courses beneath the optic nerve, adhering tightly to the optic strut's superior surface, while posteromedially, arachnoid bands tether the ICA to the Liliequist's membrane. Understanding these relationships is paramount to avoiding injury to the optic nerve (superior and medial to the rings), oculomotor nerve (inferior and lateral), and ICA itself, particularly during anterior clinoidectomy or dural peeling [5, 9].

### Description of the technique

#### Position

The patient is prone, with the head turned 30° to the opposite side of the approach and slightly extended. The head is securely fixed in a Mayfield three-pin clamp with the vertex down to facilitate access to the paraclinoid region [8].

#### Conventional pterional approach

When a standard pterional approach is selected, the initial step involves a frontotemporal skin incision as described by Yaȿargil, starting immediately anterior to the tragus and extending superiorly and anteriorly, staying within or behind the hairline to optimize cosmetic outcomes. The dissection proceeds meticulously through the subcutaneous tissues, carefully identifying and preserving the frontal branch of the facial nerve. Subsequently, the temporalis fascia is incised, and the muscle is elevated from the temporal fossa, reflecting it anteriorly and inferiorly to expose the underlying bone. Then, a frontotemporal craniotomy is performed using a high-speed drill, creating a bone flap that extends from the superior temporal line to the floor of the middle fossa, taking care to preserve the integrity of the dura mater. The craniotomy should be adequately extended to allow for sufficient access to the skull base and the region of the anterior clinoid process, which will be the target for subsequent dissection and drilling [8].

#### Pterional keyhole approach

In the case presented here, we used a sphenoid ridge keyhole approach. A 5 cm curvilinear incision is marked at the level of the hairline. The hair is shaved 1 cm behind the marked incision, and the skin is draped in a standard fashion [8]. After elevation of the skin flap, the temporal muscle is carefully split along its fibers, preserving its neurovascular supply, and reflected anteriorly without extensive detachment. A single burr hole is placed at the posterior edge of the planned craniotomy site. The bone flap is then elevated using a high-speed craniotome, with particular care taken to meticulously detach the sphenoid crest with a fine bone chisel to prevent dural injury.

#### Anterior Clinoidectomy

After the craniotomy, the procedure is performed under microscopic magnification. Following the elevation of the bone flap, the lateral portion of the greater sphenoid wing is progressively drilled using a 4 mm or 5 mm diamond burr until the meningo-orbital band is visualized. A segment of the lateral orbital wall and the lateral margin of the superior orbital fissure is removed to enhance access.

The meningo-orbital band is meticulously coagulated with bipolar forceps and sharply incised using a No. 11 scalpel blade in a layered fashion. This maneuver exposes a dissection plane between the dura propria (outer dural layer) and the lateral wall of the cavernous sinus. The dura is then carefully peeled in a posteromedial direction to fully expose the anterior clinoid process (ACP), preparing it for subsequent removal. The superolateral portion of the ACP is removed using micro-rongeurs, proceeding in a lateral-to-medial direction until the roof optic nerve is fully exposed. The optic strut is then drilled out using a 2 mm diamond burr under copious and continuous irrigation, exposing the carotid dural rings, carotid collar, and proximal ICA.

#### Cavernous sinus hemostasis

Following the anterior clinoidectomy, we can manage any minor bleeding from the cavernous sinus by directly applying fibrin glue to the bleeding point. Alternatively, we can use a saline-soaked hemostatic sponge with gentle pressure to seal venous channels without obstructing critical neurovascular structures.

#### Surgical steps for opening the carotid dural rings

**Fig. 1:**
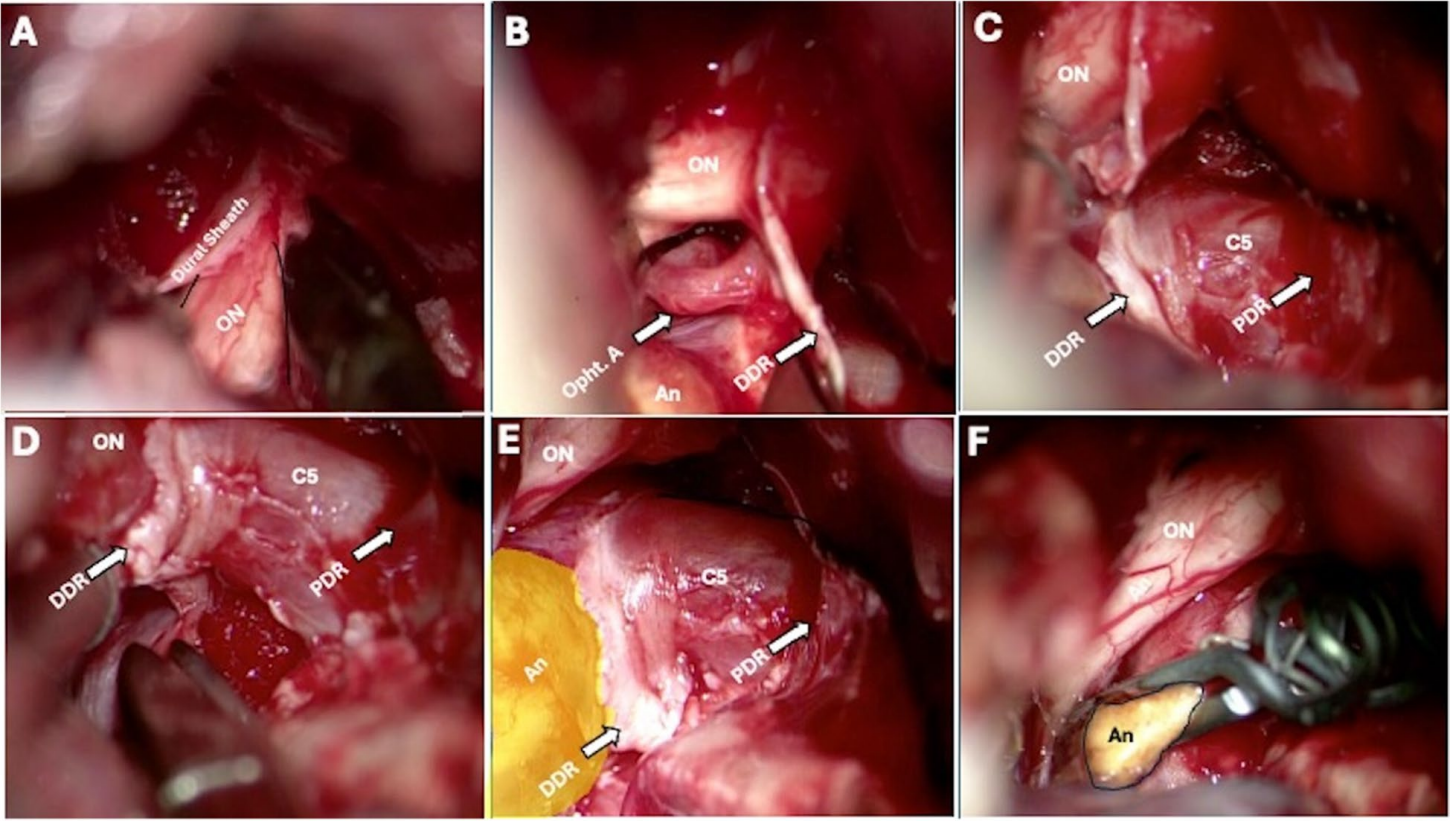
**A,** Right side conventional pterional approach. The falciform ligament and the optic nerve sheath are cut to mobilize the nerve. **B,** the position of the ophthalmic artery, the distal dural ring (DDR), and the neck of the aneurysm (An) are confirmed. **C,** the DDR is cut between the optic nerve and carotid artery, extending the cut until the medial surface of the internal carotid artery (ICA). **D,** the DDR is now sharply incised under the surface of the ICA until the medial part of the ring is reached. If necessary, at this point, some liquid hemostatic or fibrin glue is injected through the proximal ring in case of venous bleeding from the cavernous sinus. **E,** The DDR and proximal dural ring (PDR) are now exposed. The ICA can be mobilized, and the neck of the aneurysm is visualized. **F,** the aneurysm is clipped, and the anatomy is confirmed, including the patency of the ophthalmic artery (Opht. A) and the integrity of the optic nerve (ON)

***A) Dural Incision and Initial Exposure***. The dura is incised with a linear incision extending towards the optic nerve at the proximal third of the Sylvian fissure. The dura is cut along the falciform ligament and optic sheath until the anterior floor bone is reached. (Fig. 1). Micro-scissors are used to minimize trauma to the underlying neurovascular structures.

***b) Identification of Key Anatomical Structures***. Carotid arteries. The ICA is meticulously identified distal to the anterior clinoid process, with precise attention to its spatial relationship with the optic nerve, oculomotor nerve, and surrounding arachnoid membranes.

- *Oculomotor nerve (CN III).* The oculomotor nerve is identified as traversing the cavernous sinus's roof, with meticulous care devoted to preserving its structural and functional integrity throughout the dissection of its adjacent membrane to avoid traction injury.
- *Carotid dural rings.* The proximal and distal dural rings and their relationship to the anterior clinoid process are identified. These structures serve as critical landmarks for the subsequent procedural steps. Careful dissection is essential to avoid injury to the surrounding structures.
- *Proximal Control and Initial Ring Incisions.* Posteriorly, at the junction of the DDR and the proximal dural ring, where the tip of the ACP was located, a Rhoton dissector is used to gently dissect and release the carotid rings at the concave surface of the ICA, allowing the insertion of a temporary clip for proximal control (Fig. 1-C). A temporary clip is placed on the clinoid segment of the ICA to confirm that it can be safely applied, and then it is retired to be used just in case it is necessary (Figure 3).
- *Identification of the Ophthalmic Artery.* The optic nerve is gently retracted superomedial, and the DDR is retracted inferolateral to identify, dissect, and protect the ophthalmic artery up to its origin (Fig. 1-B). Identifying the ophthalmic artery before opening the carotid rings can prevent vascular injury. However, it is essential to remember that anatomical variations may be present.

***c) Lateral DDR Incision.*** The lateral aspect of the DDR is sharply incised using micro-scissors, retracting the inferior dural membrane ventrally (Fig. 1-D).

***d) Superior And Inferior DDR Release. Superior incision.*** Small and sharp cuts are progressively performed at the superior aspect of the DDR, extending towards the lateral wall of the sphenoid sinus (Fig. 1-C).

*Inferior incision.* The inferior aspect of the DDR is freed from the ICA's concave surface, maintaining a single-layer dissection plane (Fig. 1-E). The incision is made with a series of minor, careful cuts parallel to the ICA axis, advancing toward the lateral wall of the sphenoid sinus.

***e) Final Mobilization and Verification***. *Complete DDR release.* A Rhoton dissector is used to mobilize the freed dural ring, confirming 360-degree circumferential exposure of the internal carotid artery.

*Aperture verification.* The adventitial surface of the internal carotid artery and the optic strut region are thoroughly inspected to ensure no residual fibrous bands remain.

***f) Microsurgical Clip Reconstruction.*** Meticulous microsurgical techniques are typically employed to precisely apply a microsurgical clip across the neck of the aneurysm. The carotid artery can be mobilized if necessary to obtain the optimal vision of the neck and dome.

***g) Dural closure.*** The dura is meticulously closed using a running suture technique with a 4–0 or 5–0 Prolene suture. In cases where the ACP is pneumatized, a fat graft is harvested and placed together with fibrin glue in the space created by the anterior clinoidectomy at the level of the optic strut to prevent CSF leakage.

### Indications

*Paraclinoid aneurysms.* This is the primary indication for opening the DDRs, as it allows for proximal control, mobilization of the ICA, and facilitation of clip application, including incidental and symptomatic paraclinoid aneurysms.

*Intracavernous aneurysms.* Opening the carotid dural rings together with the middle fossa peeling facilitates the surgical clipping or wrapping of intracavernous aneurysms and bypass procedures.

### Limitations

*Post-Surgical or Post-Radiosurgical Anatomy.* Prior interventions (e.g., craniotomy, radiosurgery) may cause fibrosis, adhesions, or distortion of tissue planes; this may complicate the identification of the carotid dural rings, optic nerve, or ICA. Scarring from previous surgery can obscure the meningo-orbital band, carotid collar, or arachnoid cisterns, increasing the risk of iatrogenic neurovascular injury [2]. The angioarchitecture should be assessed preoperatively to detect pneumatization of the ACP, interclinoid bridge or caroticoclinoid ring, because these anomalies make it difficult to carry out the extradural clinoidectomy. In cases where the aneurysm erodes the anterior clinoid process, the clinoidectomy should be performed intradurally to reduce the risk od intraoperative rupture.

### How to avoid complications

*Preoperative planning.* Preoperative planning with 3D angiography (CTA/MRA/DSA) is highly advised to assess aneurysm morphology and direction, ICA tortuosity, and the relationships to the optic nerve, anterior clinoid, and cavernous sinus as well as identification of anatomic variants (e.g., extradural ophthalmic artery, carotid-clinoid foramen, etc.) [4].

*Proximal control.* In giant or big paraclinoid aneurysms, proximal control should be obtained before making incisions in the DDR. This proximal control can be achieved at the clinoid segment of the ICA after retiring the clinoid process, the petrous carotid, the cervical segment of the ICA, or through endovascular balloon occlusion.

After the final clip application, a fluorescein videoangiography is recommended to confirm complete aneurysm obliteration and patency of all adjacent vessels [4].

Regarding dissection security, cutting the DDR should be performed with blunt-tipped microscissors, maintaining a dissection plane parallel to the ICA axis, and using short, controlled cuts (Fig. 1-C-D).

*Optic Nerve Protection.* The optic nerve dural sheath should be sharply incised along its dorsal aspect to enable the safe mobilization of the nerve while exposing the ophthalmic artery. The use of continuous and abundant saline irrigation when drilling the anterior clinoid process is advisable. Avoid the drill whenever possible, favoring the use of small-tip rongeurs.

*CSF Prevention.* A watertight dural closure should be achieved to avoid CSF. If a watertight dural closure is not possible, we use a vascularized pericranial flap, a synthetic dural substitute, and fibrin glue. The optic strut should be inspected to identify and seal any potential communication between the sphenoid sinus and subarachnoid space. Fat grafts or muscle packing can be strategically employed for this purpose.

*Cavernous sinus hemostasis.* If there is bleeding from the cavernous sinus, we can use small pieces of soaked hemostatic sponge and cottonoids to compress the bleeding point. Alternatively, fibrin glue or another hemostatic agent can be applied to the cavernous sinus to control the bleeding. Caution should be exercised when using bipolar coagulation near cranial nerves III and IV to avoid thermal injury.

Postoperative Measures. Postoperative monitoring is crucial, as the patient should be closely observed in an intensive care unit for at least 24–48 h after the surgery. This surveillance includes regular neurological assessments, continuous vital sign monitoring, and prompt management of potential complications, such as vasospasm, intracranial hypertension, or seizures. Conducting a CT angiography or DSA within 24 h is advisable to rule out any ICA stenosis or residual concerns.

### Specific information to patient

Standard preoperative neurosurgical workup and patient consent should be performed. The potential complications, including bleeding, infarction, infection, cerebrospinal fluid fistula, and neurological deficits, should be discussed with the patient [3]. Given the nature of the keyhole approaches, even when the deep surgical field is the same as the standard pterional approach, there is a potential risk when managing the carotid artery, optic nerve, and oculomotor nerve. The patients should have a pre- and postoperative evaluation of their visual function and movements.

The treatment approach for each patient should be determined through a multidisciplinary collaboration between the endovascular and microsurgical teams. The decision should be based on the collective expertise and outcomes of both groups within the hospital [1].

## Conclusions

As microsurgery has continued to develop and advance, the principal objective has been to reduce patient risks and achieve the best outcomes, particularly after surgeries for paraclinoid aneurysms where endovascular procedures present a high rate of recurrence. Opening the carotid dural rings is widely regarded as a challenging procedure for neurosurgeons. Therefore, a meticulous, stepwise dissection and thorough understanding of the regional anatomy are essential to achieve good outcomes (1, 8).

## Supplementary Information

Below is the link to the electronic supplementary material.

Supplementary file1 (MP4 44497 KB)

## Data Availability

No datasets were generated or analysed during the current study.
